# Supplementary material for: CamoTSS: analysis of alternative transcription start sites for cellular phenotypes and regulatory patterns from 5' scRNA-seq data
Source: Nat Commun. 2023 Nov 9;14:7240. doi: 10.1038/s41467-023-42636-1 (PMC10636040; doi:10.1038/s41467-023-42636-1)
Supplement: Supplementary file 1 — Supplementary Information [file 41467_2023_42636_MOESM1_ESM.pdf]

Supplementary Figures for "CamoTSS: analysis of alternative transcription start sites for cellular phenotypes and regulatory patterns from 5' scRNA-seq data"

Ruiyan Hou, Chung-Chau Hon and Yuanhua Huang*

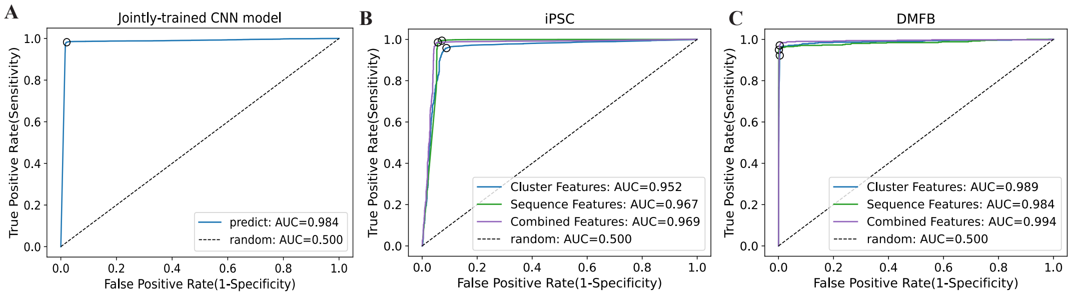

Figure S1: Prediction performance with different models, features and datasets. (A) Receiver operating characteristic curve for prediction of TSS clusters when combining reads-based (i.e., cluster features) and sequence-based features with joint training by concatenating the four reads-based features to the second last layer of CNN model. Shown is based on the combined dataset with iPSC and DMFB, same as main Fig. 1D. (B,C) ROC curves for iPSC (B) and DMFB (C) datasets with different feature groups by using logistic regression models. Panel B and C have the same model setting as main Fig. 1D.

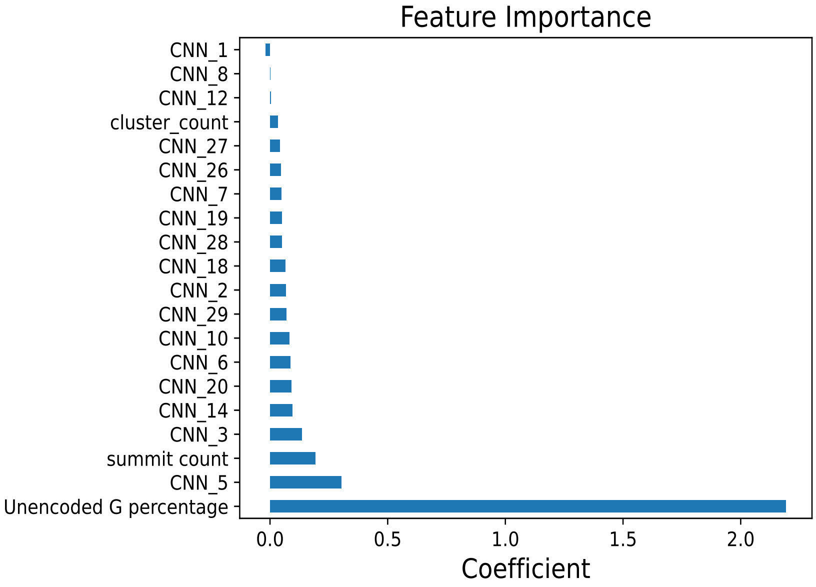

Figure S2: Bar plot showing feature importance ranking based on the coefficient in the logistic regression. All features were standardized at first.

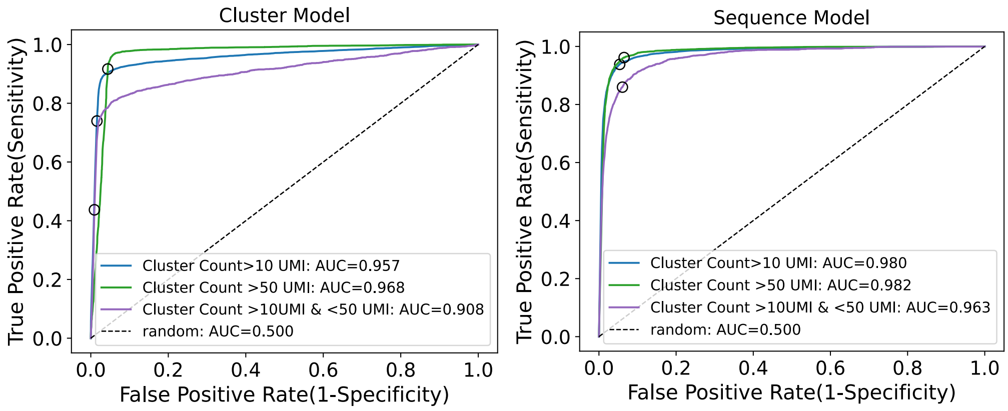

Figure S3: ROC curves for different datasets splited by cluster counts (i.e. 10 UMI and 50 UMI) and predicted by cluster model (left panel) and sequence model (right panel). Ten-fold cross-validation is used for the evaluation.

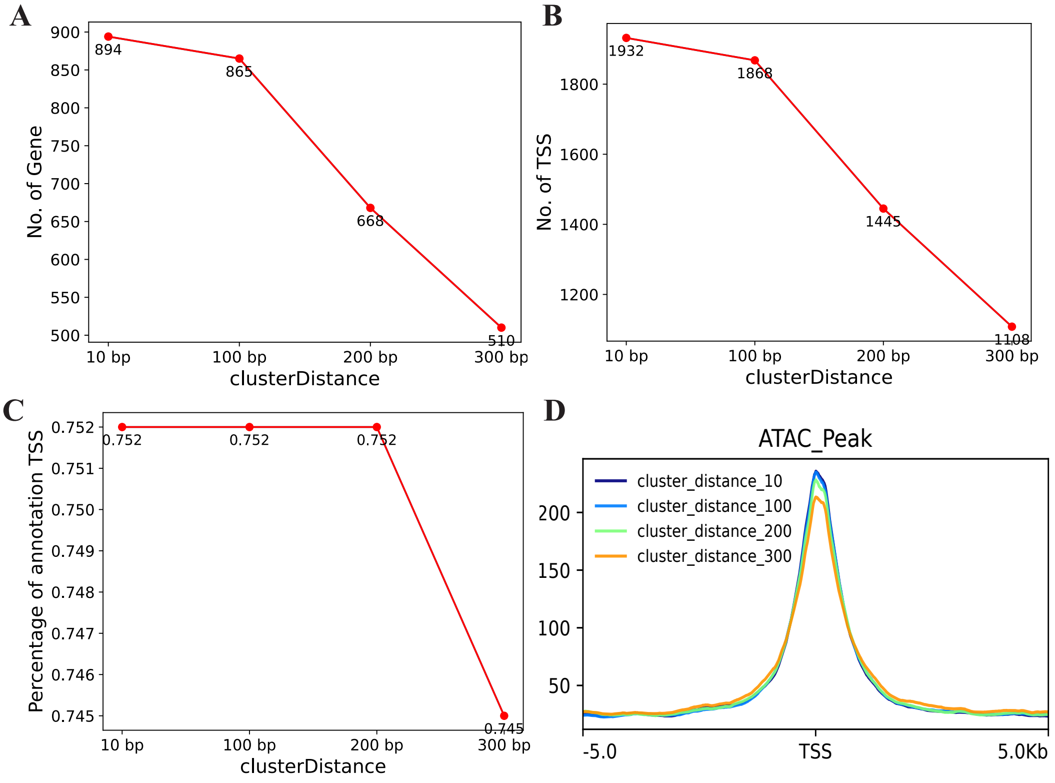

Figure S4: Evaluation of different inter-cluster distances. Line chart showing the number of genes with alternative TSS (A) and TSS (B) and percentage of annotated TSS (C) detected by CamoTSS in PBMC dataset. Peak plot displaying the scATAC-seq signal around TSS detected based on various inter-cluster distances (D).

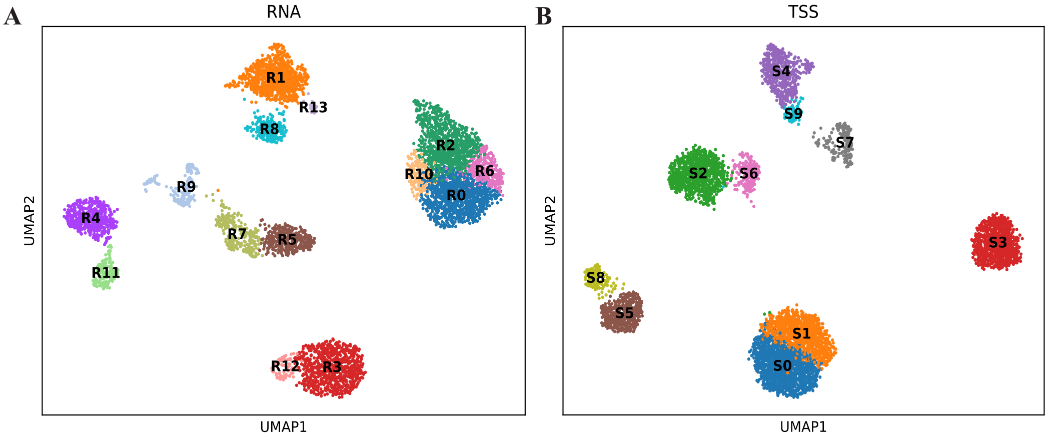

Figure S5: Uniform Manifold Approximation and Projection (UMAP) representation of 5732 single-cell transcriptomes clustered by gene expression (A) and TSS expression (B), colored by their according cell types. Prefix 'R' and 'S' denotes cell clusters of RNA and TSS, respectively.

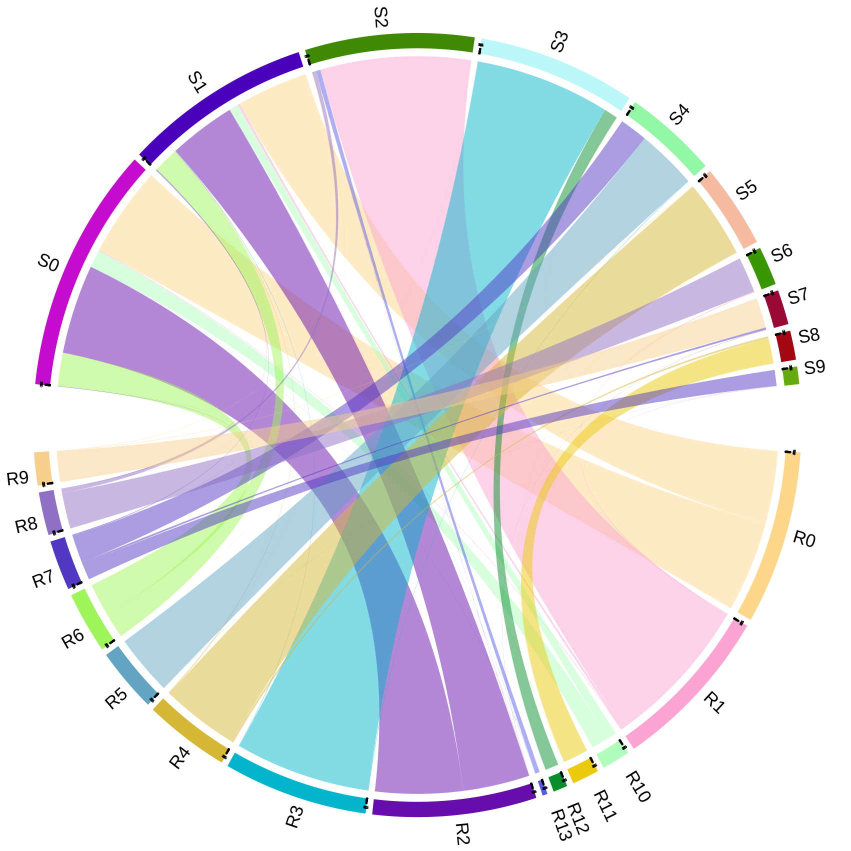

Figure S6: Chord diagram showing the relationship between all RNA-based (i.e., gene-level) clusters and all TSS-based clusters. Cluster IDs are the same as main Fig. 2A.

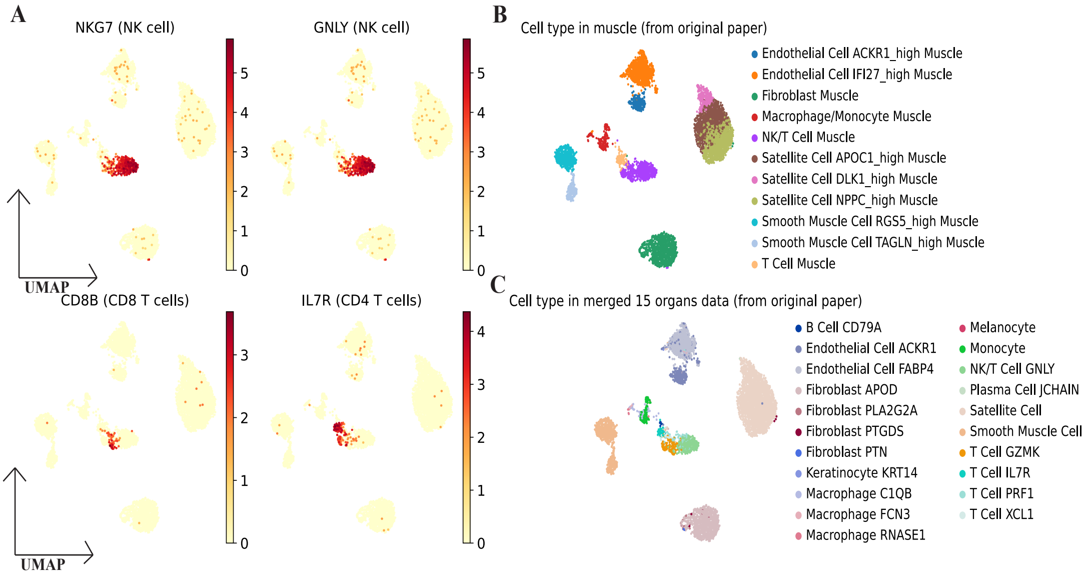

Figure S7: UMAP projections of the 15 organs dataset colored according to (A) the expression of NKG7, GNLY, CD8B and IL7R, (B) cell type annotation from the original paper on individual organ (muscle) with lower resolution, and (C) cell type annotation from the original paper by pooling cells from all organs with higher resolution.

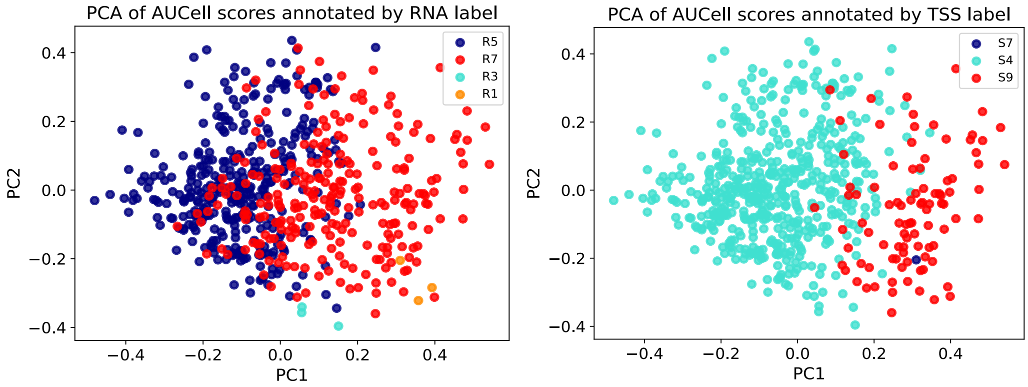

Figure S8: Scatter plot showing the top2 PCs when doing PCA for the AUCell scores. Dots were colored by RNA-level labels (left panel) and TSS-level labels (right panel), respectively.

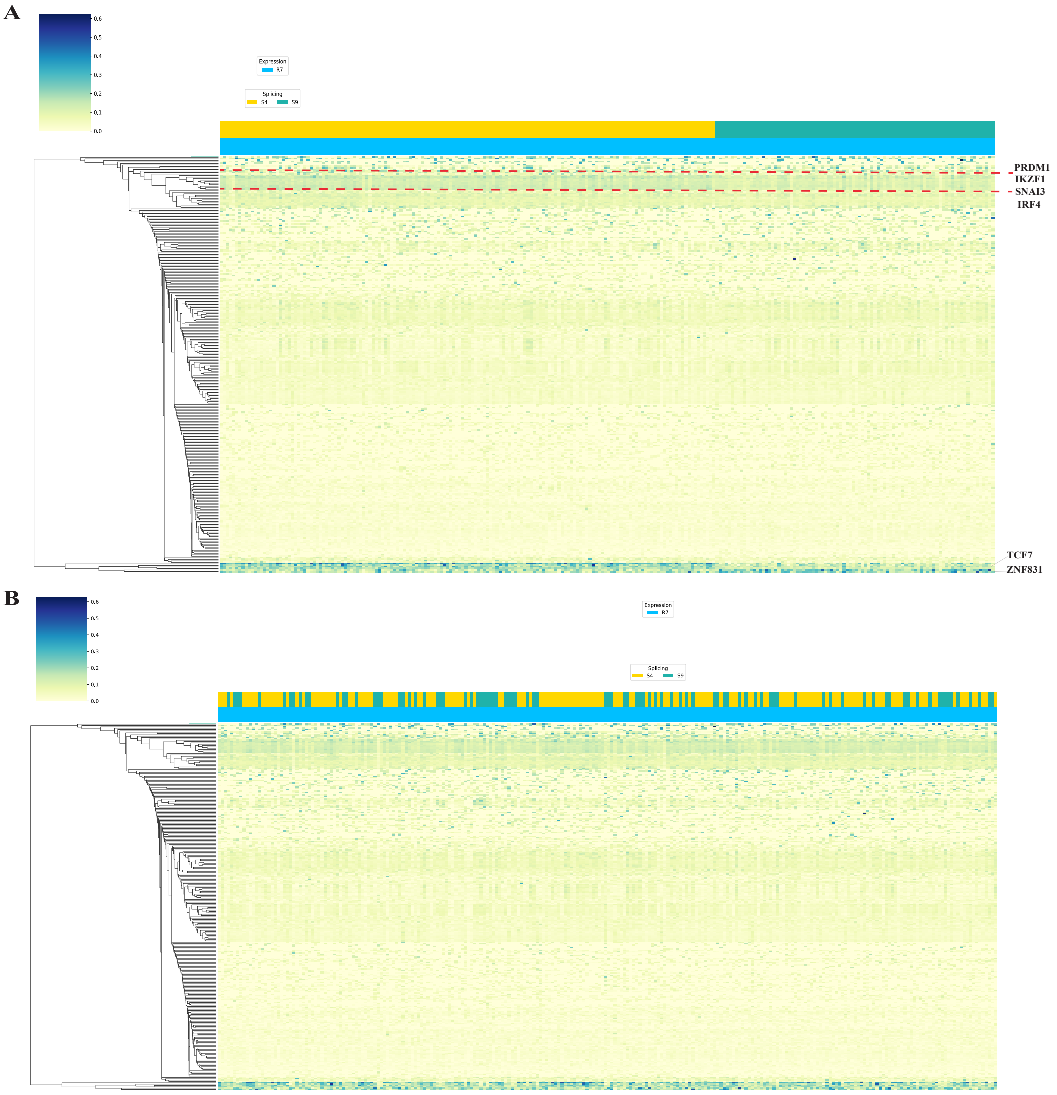

Figure S9: Heatmap showing SCENIC analysis of regulon (TF) activity in the R7 cluster of cells with S4 and S7 order (A) and random order (B).

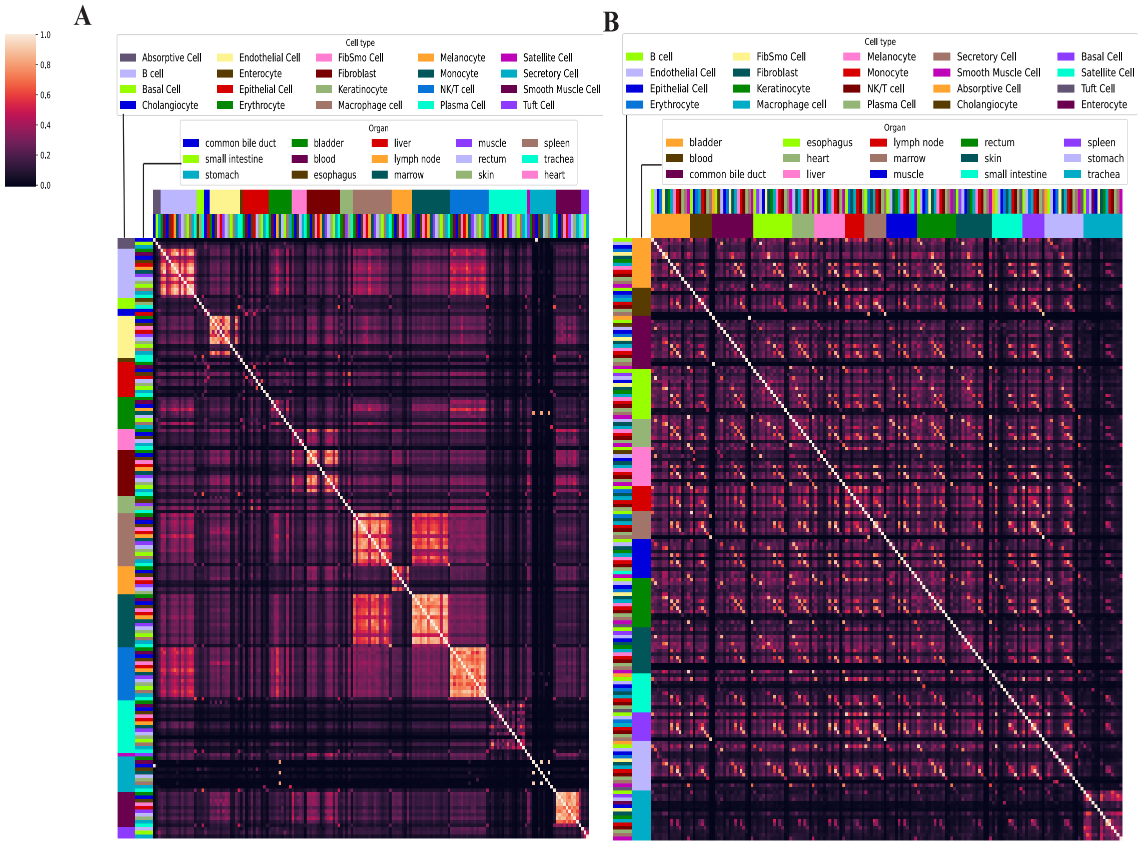

Figure S10: Heatmaps based on Pearson's correlation coefficient of profile of all cell types and sorted by organs (A) and cell types (B).

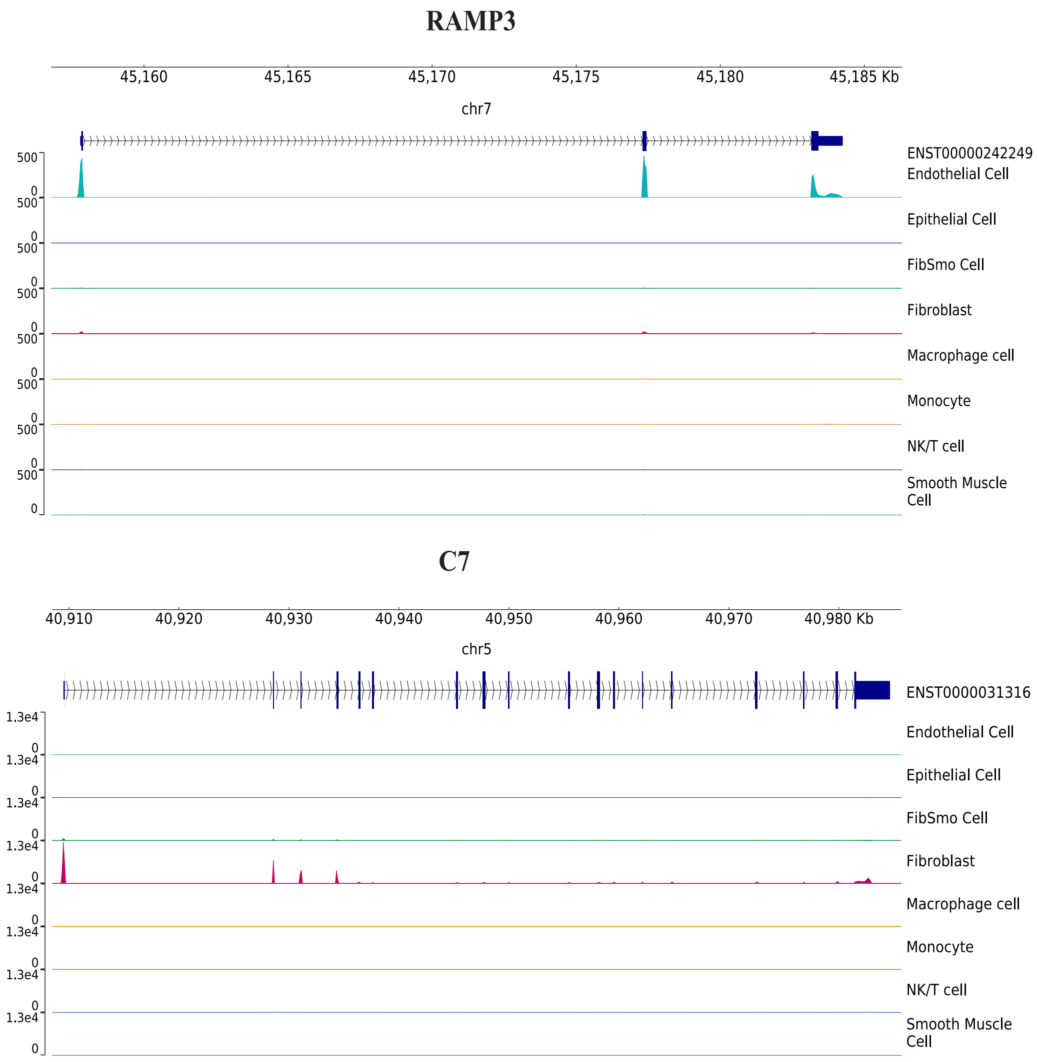

Figure S11: Track plots for genes in Fig. 3F (i.e. RAMP3 and C7). The structure of genes is shown in UCSC style. The peak showing the coverage of different cell types in scRNA-seq.

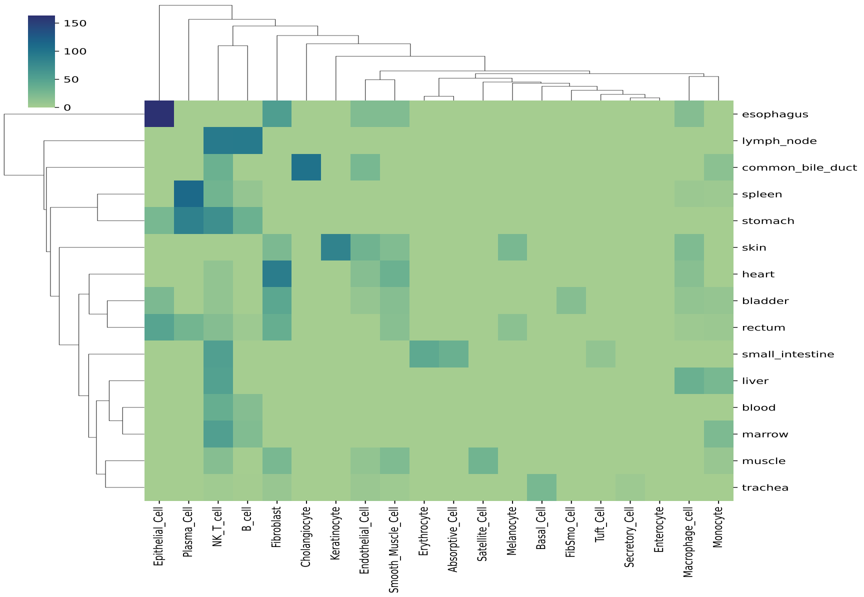

Figure S12: Heatmap showing the number of genes which have at least one TSS with a significantly differential ratio between one cell type vs all other cell types, across 15 organs

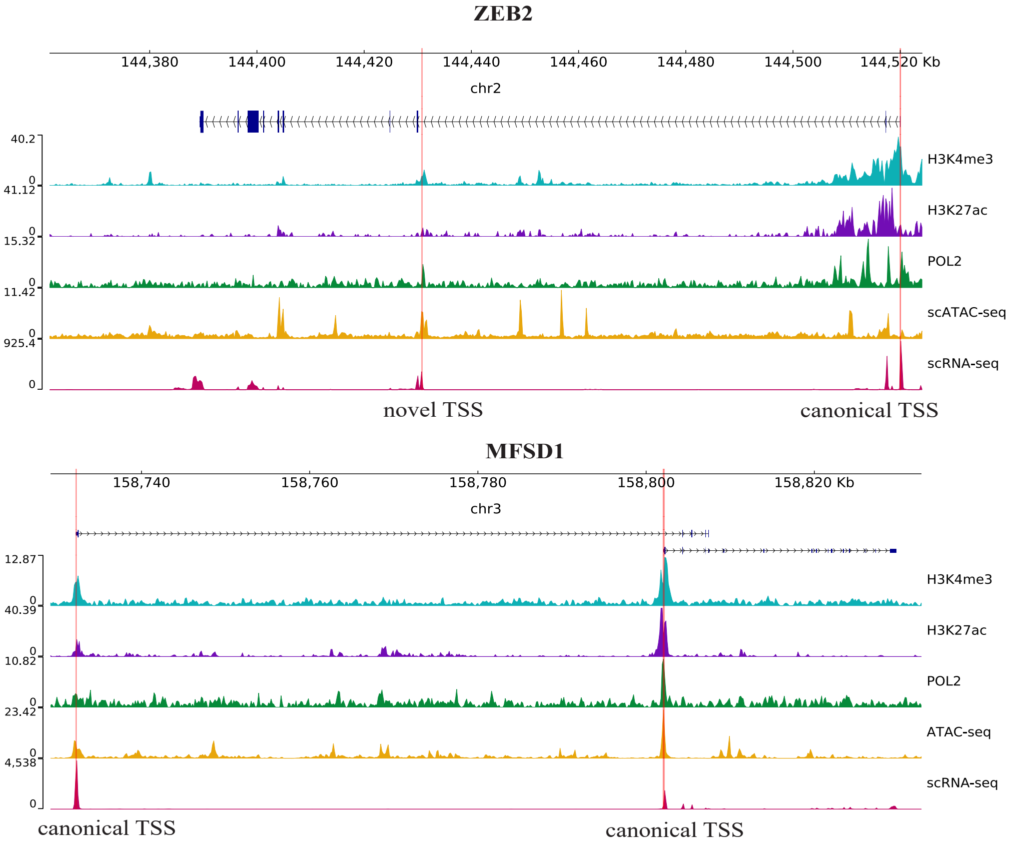

Figure S13: Tracks plot showing the coverage of histone modification mark (i.e H3K4me3 and H3K27ac), RNA POL2 and ATAC-seq of ZEB2 (Top) and MFSD1 (Bottom) in muscle and heart, respectively. The structure of genes is shown in UCSC style. The red line highlights the TSS detected by CamoTSS.

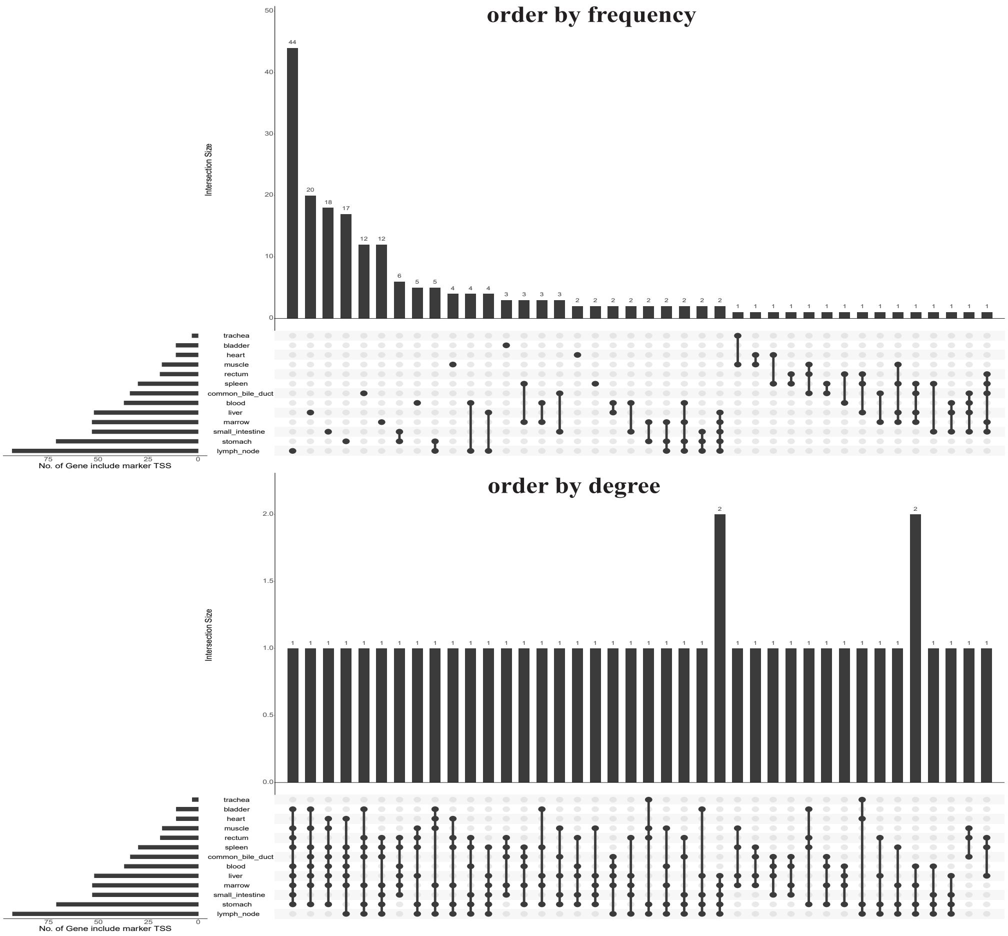

Figure S14: Upset plots displaying intersection of TSS marker in 13 organs. The upper panel is ordered by frequency. The bottom panel is ordered by the degree of intersection.

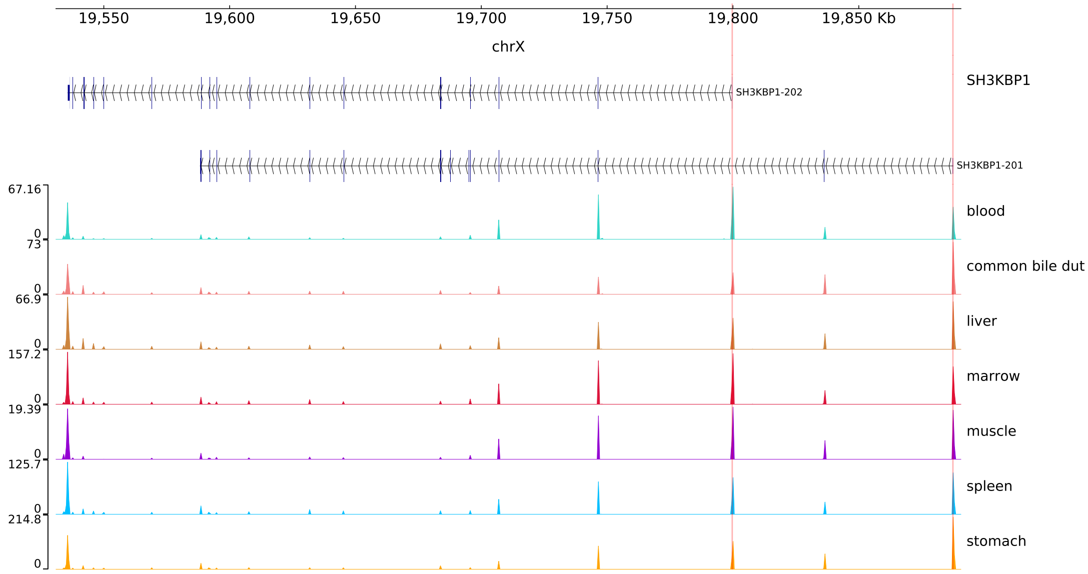

Figure S15: Tracks plot showing the coverage of SH3KBP1 in different organs in scRNA-seq data. Figure format is the same as Supp. Fig. S6.

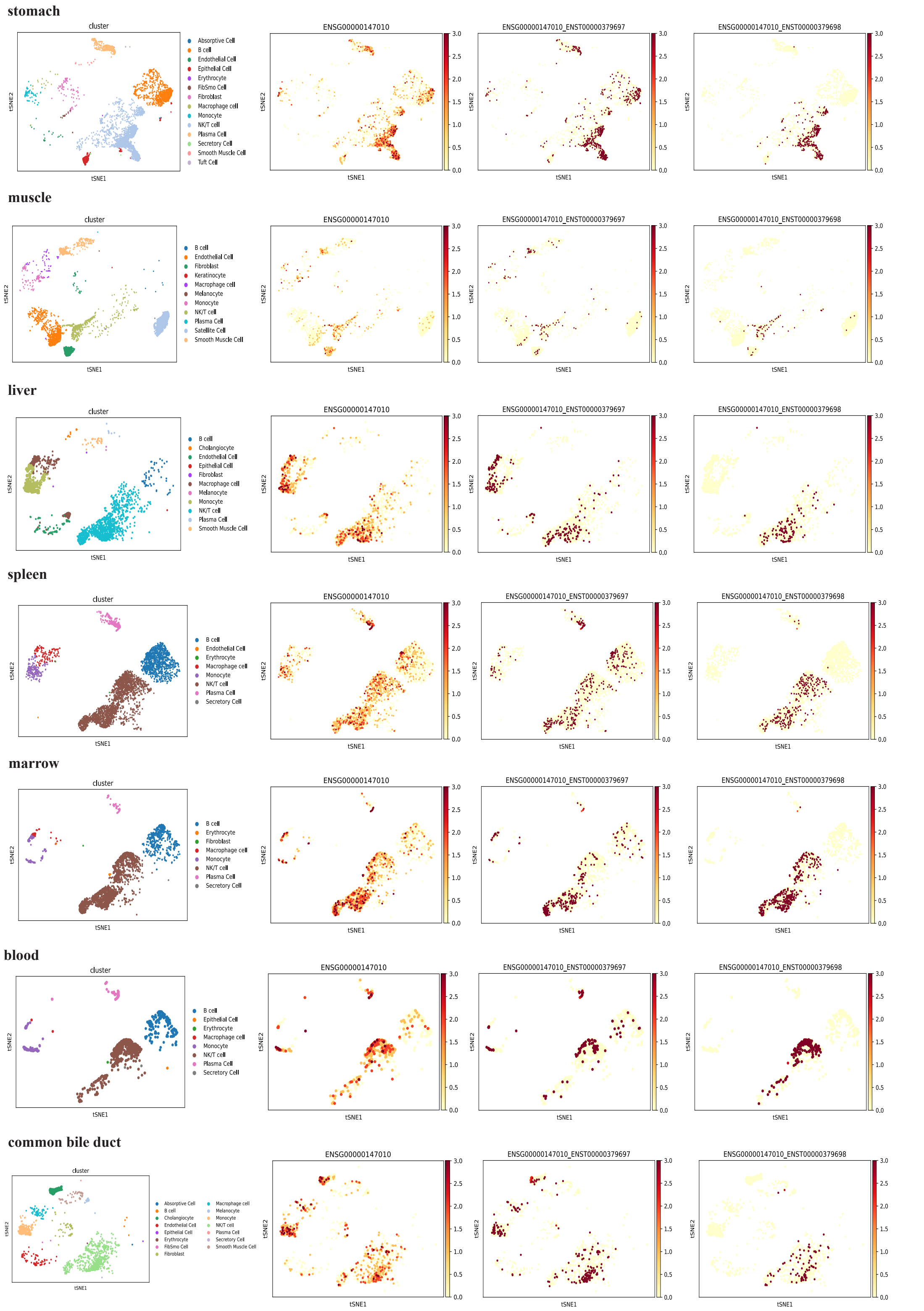

Figure S16: tSNE visualization showing cell clusters of different 7 organs (left panel). The SH3KBP1 gene-level expression (middle panel) and two SH3KBP1 TSSs (right panel) distribution in log1p(count) across cells superimposed on the t-SNE of the cells in 7 different organs.

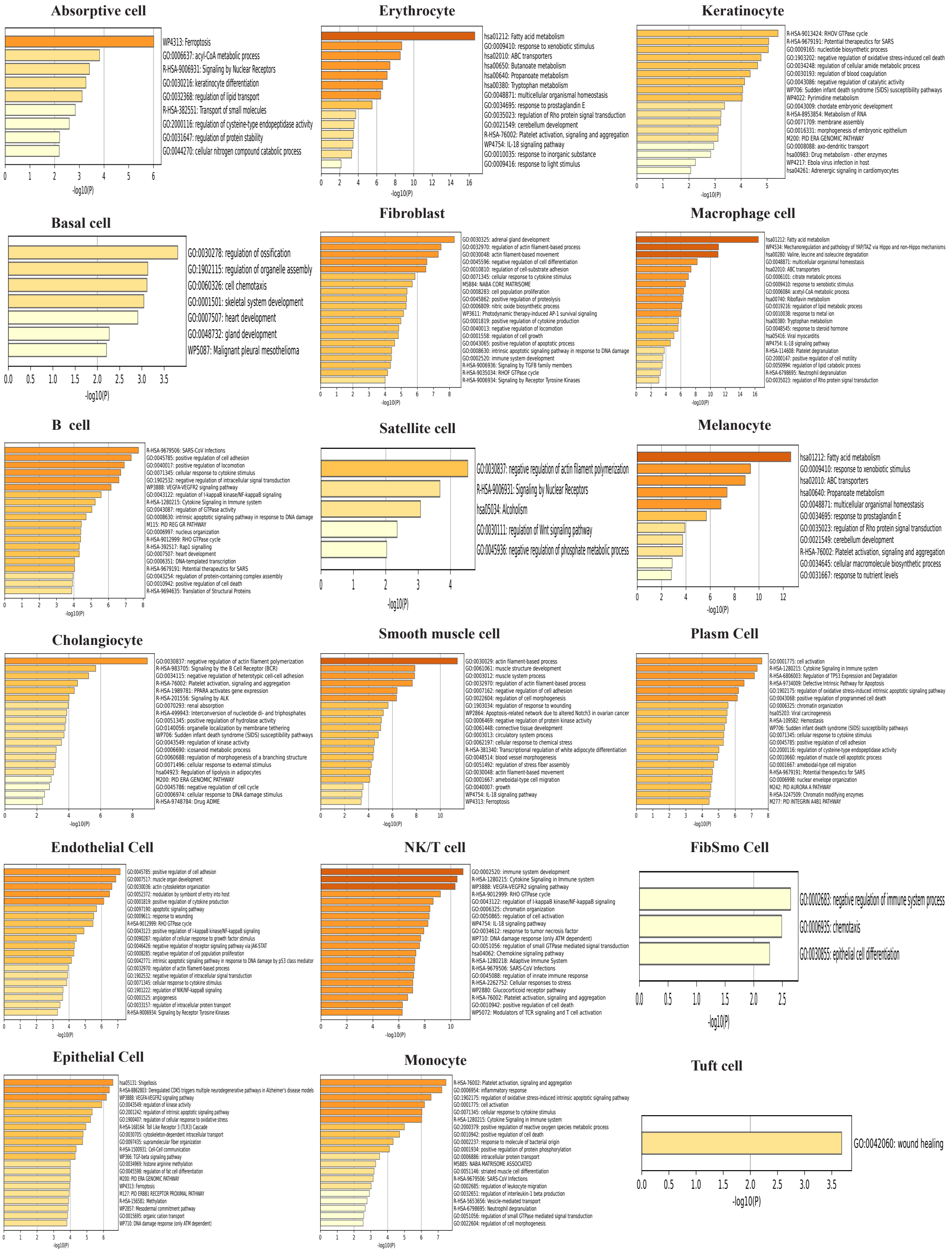

Figure S17: Bar plots of enriched GO terms over genes with significant alternative TSS usage.

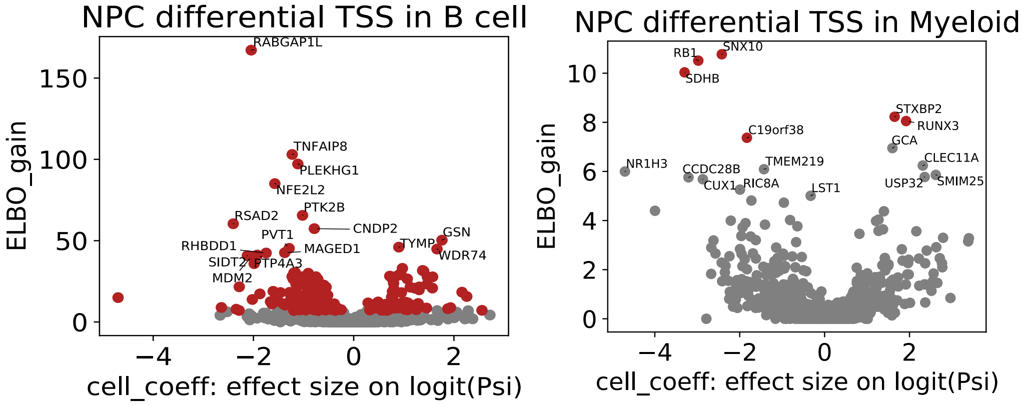

Figure S18: Volcano plots between ELBO_gain and effect size on logit(PSI) for detecting differential TSS usage between NLH and NPC patients, in B cells (left panel) and Myeloid cells (right panel). PSI value denotes the proportion of TSS1 between two major TSSs. Source data are provided as a Source Data file.

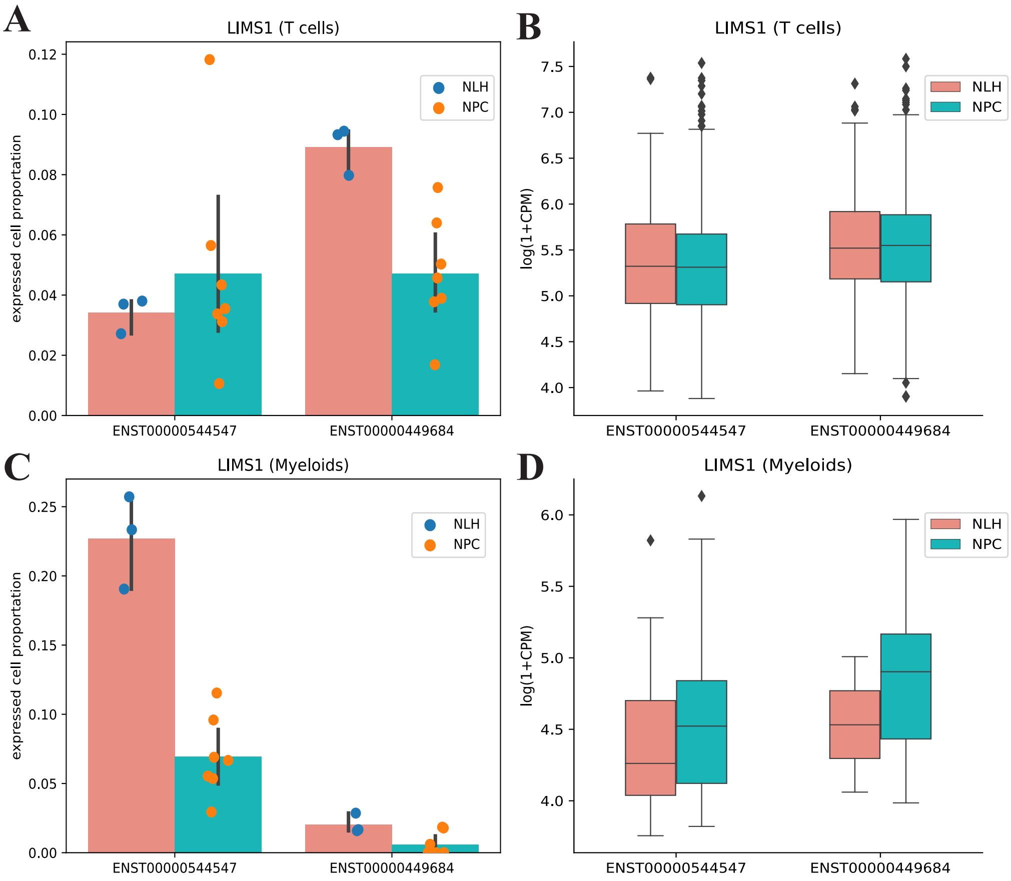

Figure S19: Expression profile of TSSs of LIMS1 in T cells and Myeloids. (A,C) Bar plot showing the expressed cell proportion of two alternative TSS of one gene between NLH and NPC patients, in T cells (A) and Myeloid cells (C). Box plots (B,D) showing the expression value of LIMS1 in the expressed cells in T (B) and Myeloid cells (D), corresponding to (A) and (C).

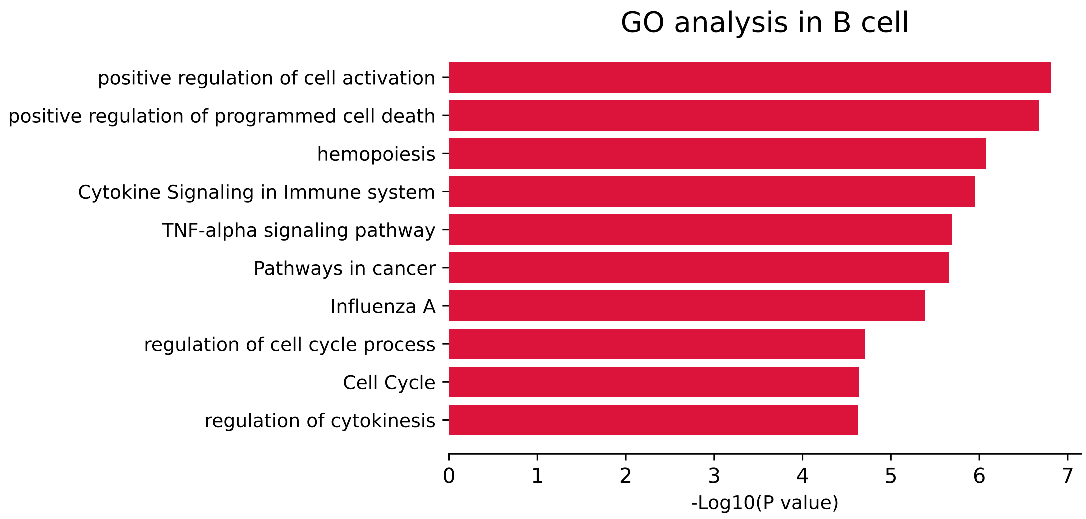

Figure S20: Bar plot showing the enriched terms of genes with differential TSS usage between NLH and NPC patients in B cells. Source data are provided as a Source Data file.

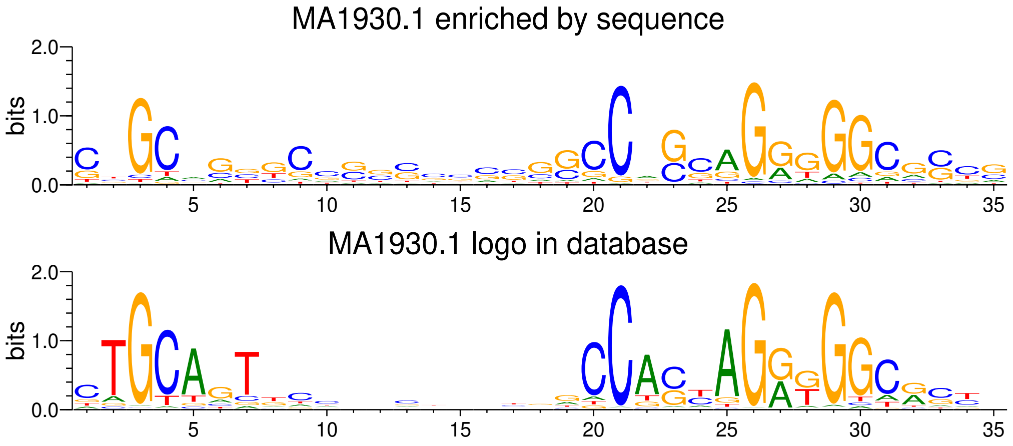

Figure S21: WebLogo of the base frequency of MA1930.1 (i.e. one motif of CTCF) enriched in the sequences detected by FIMO (top) and displayed in the JASPAR database (bottom).

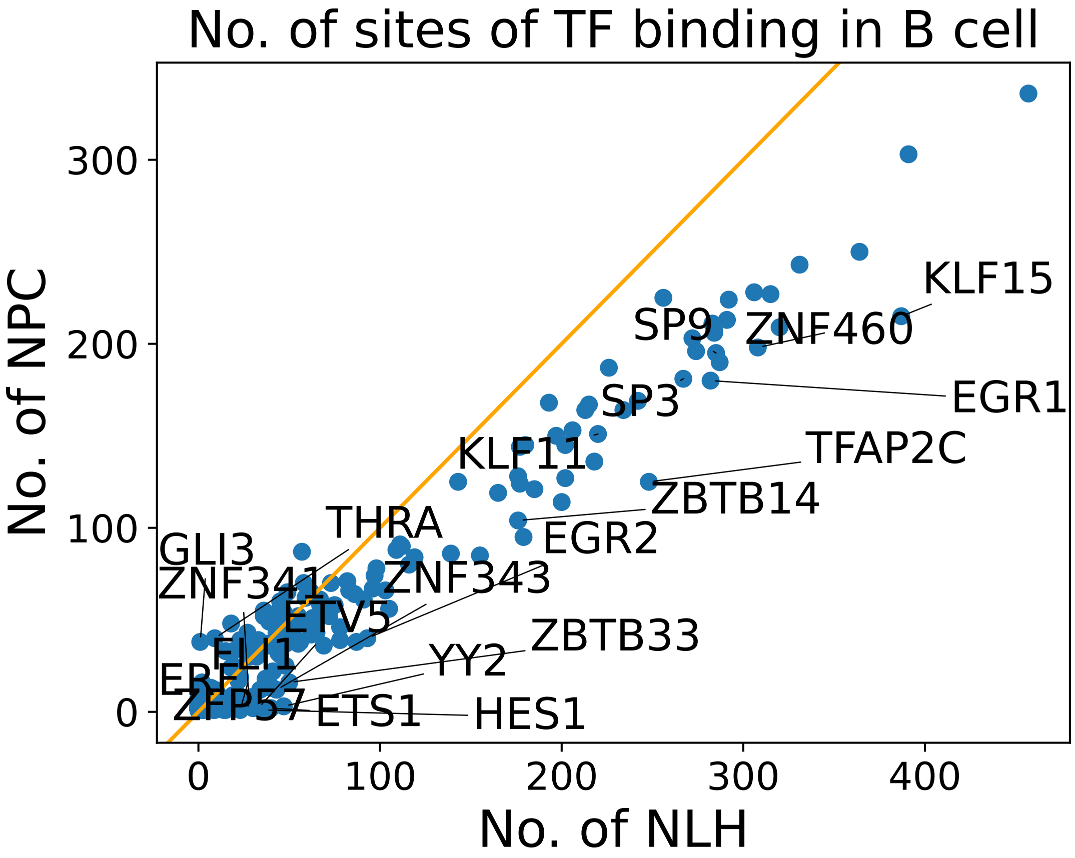

Figure S22: Binding frequency in different scenarios. (A) Scatter plot showing the binding frequency of human TFs detected by FIMO in B cells of NLH and NPC patients. (B) Scatter plot showing the binding frequency of human TF with two equal-sized random sequence sets of TSSs in NPC dataset.

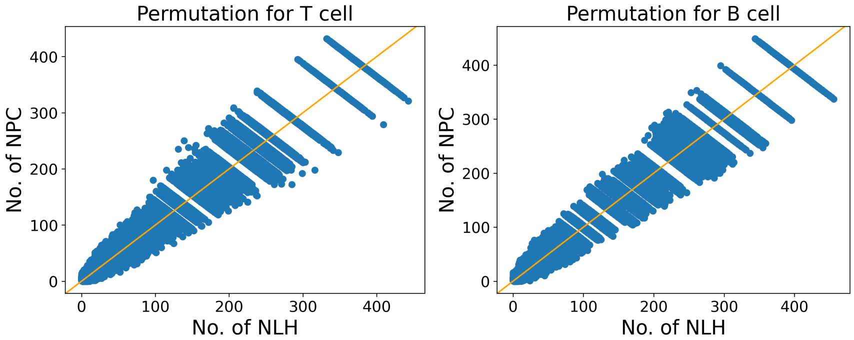

Figure S23: Scatter plot showing the binding frequency of human TF when swap randomly the NLH-preferred TSS and the NPC-preferred TSS of one gene in T cell (left panel) and B cell (right panel).

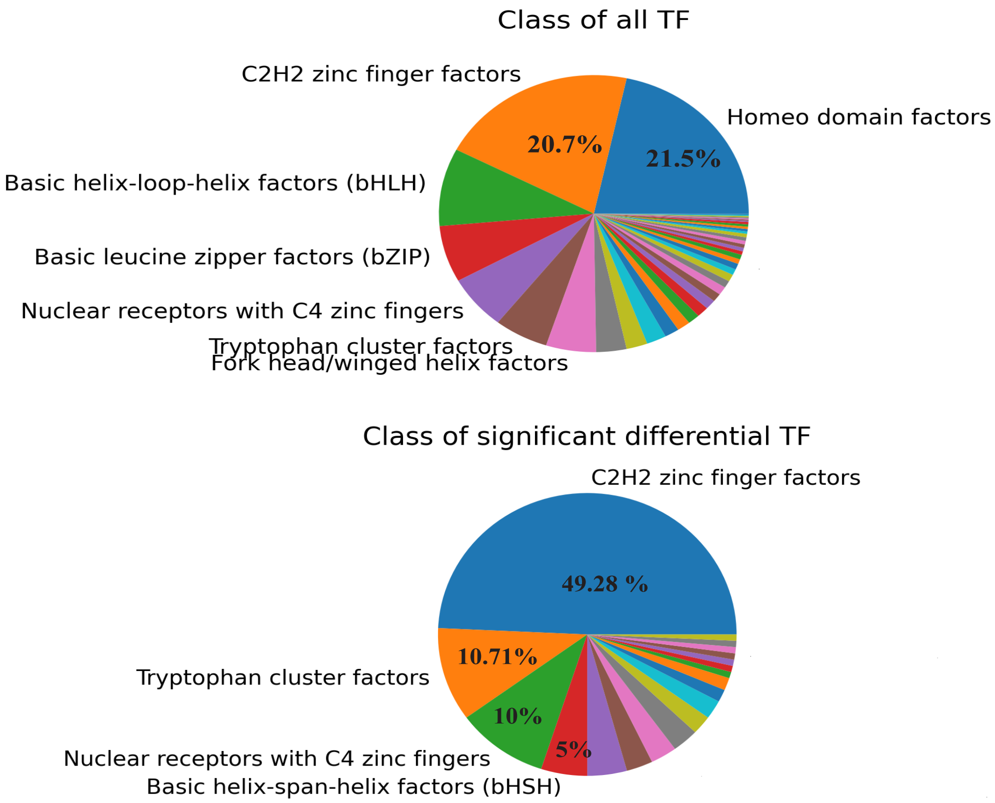

Figure S24: Pie chart showing the percentage of distinct classes of all TFs analyzed by FIMO (top panel). Pie chart showing the proportion of significant differential TFs detected by FIMO between NLH and NPC patients (Fisher's exact test).

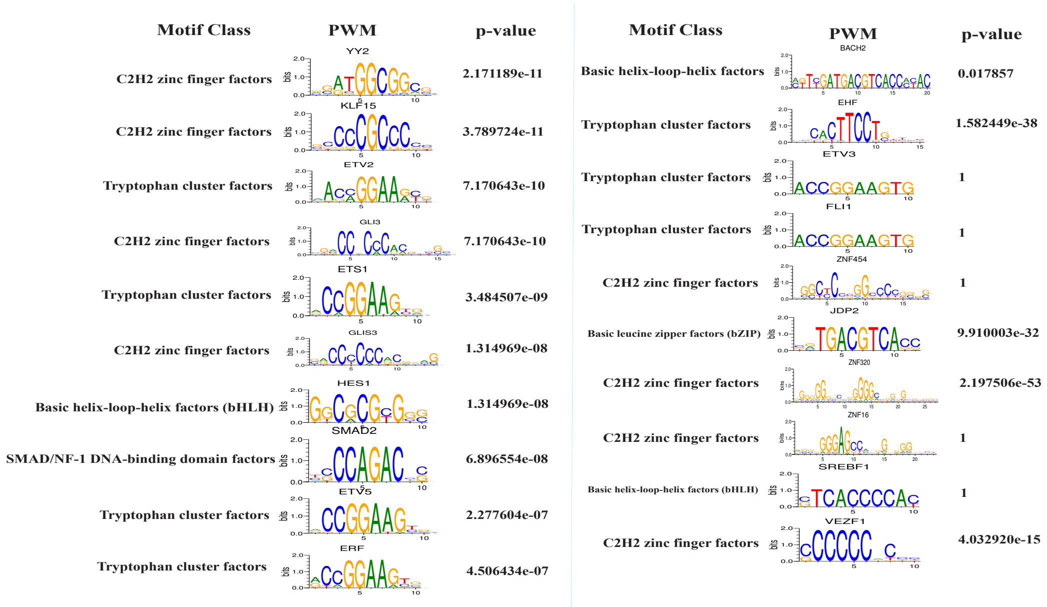

Figure S25: Weblogo showing the top 10 differential motifs and 10 randomly picked motifs.

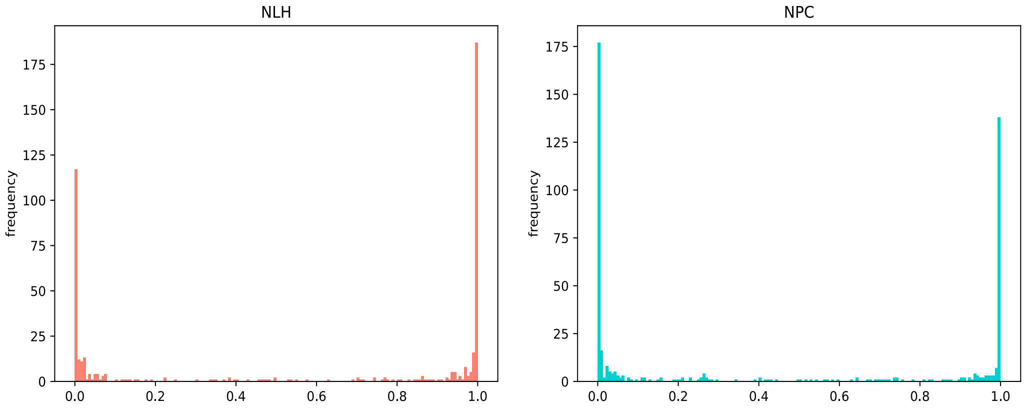

Figure S26: Histogram of probability of being positive samples predicted by our sequence-based CNN model, for the 528 TSS sequences elevated in NLH (left panel) and NPC (right panel).

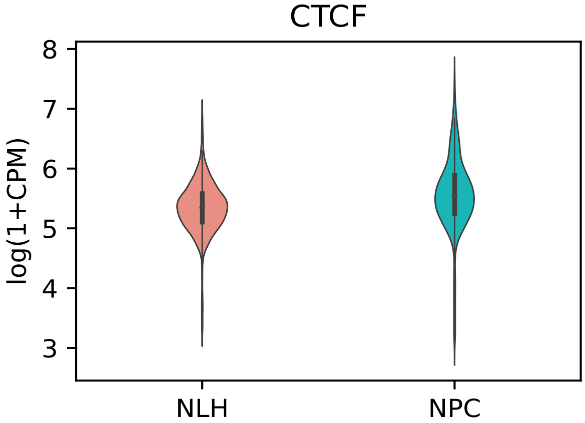

Figure S27: Violin plot showing the expression value of CTCF in the expressed cells between NLH and NPC patients.

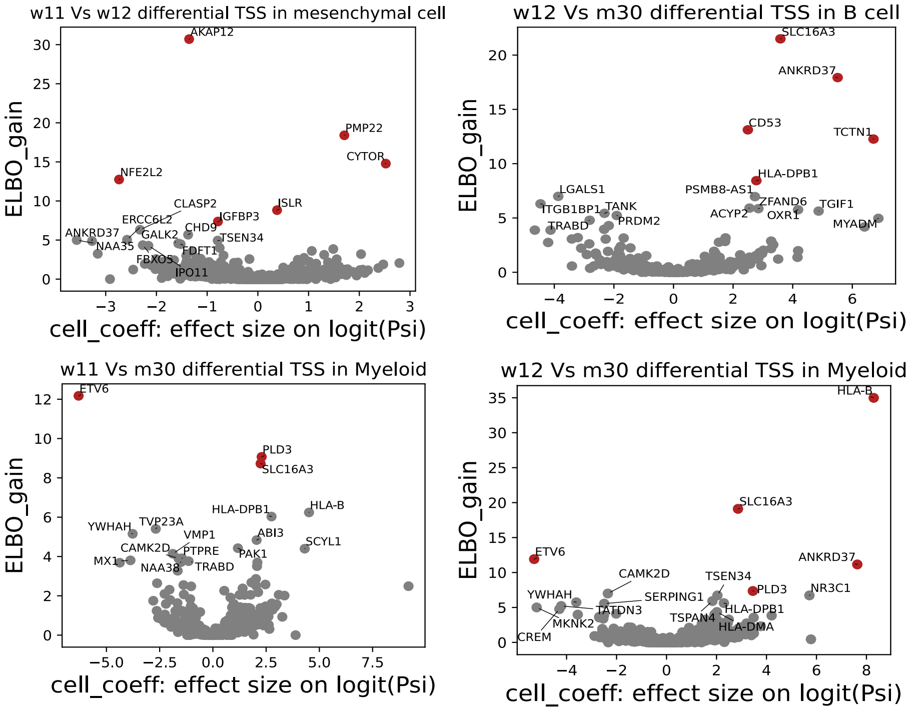

Figure S28: Volcano plots showing the relationship between ELBO_gain and effect size on logit(PSI) for all TSSs which can be detected by BRIE2. The PSI value denotes the proportion of TSS1 among the two major TSSs. Source data are provided as a Source Data file.

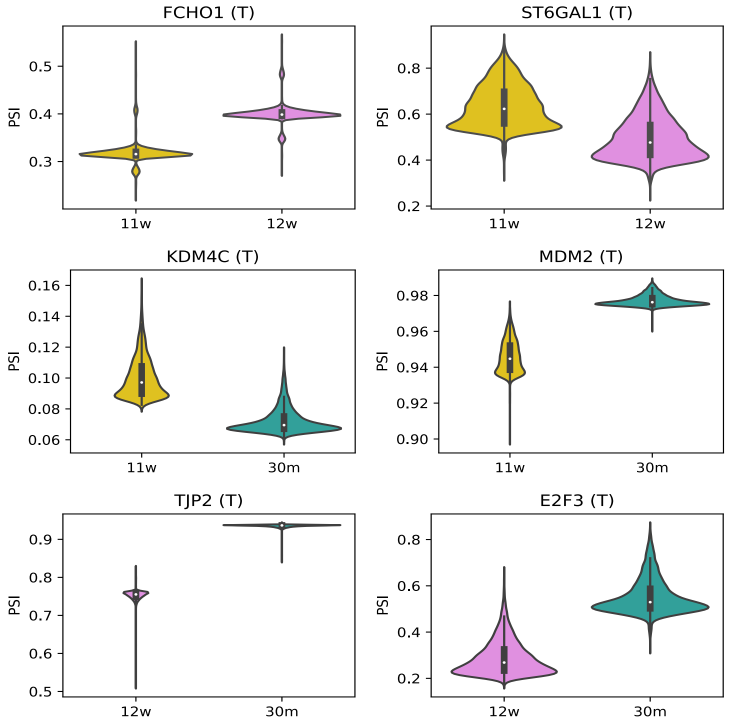

Figure S29: Violin plots of the significantly differential genes with alternative TSS usage but only between two development stages.

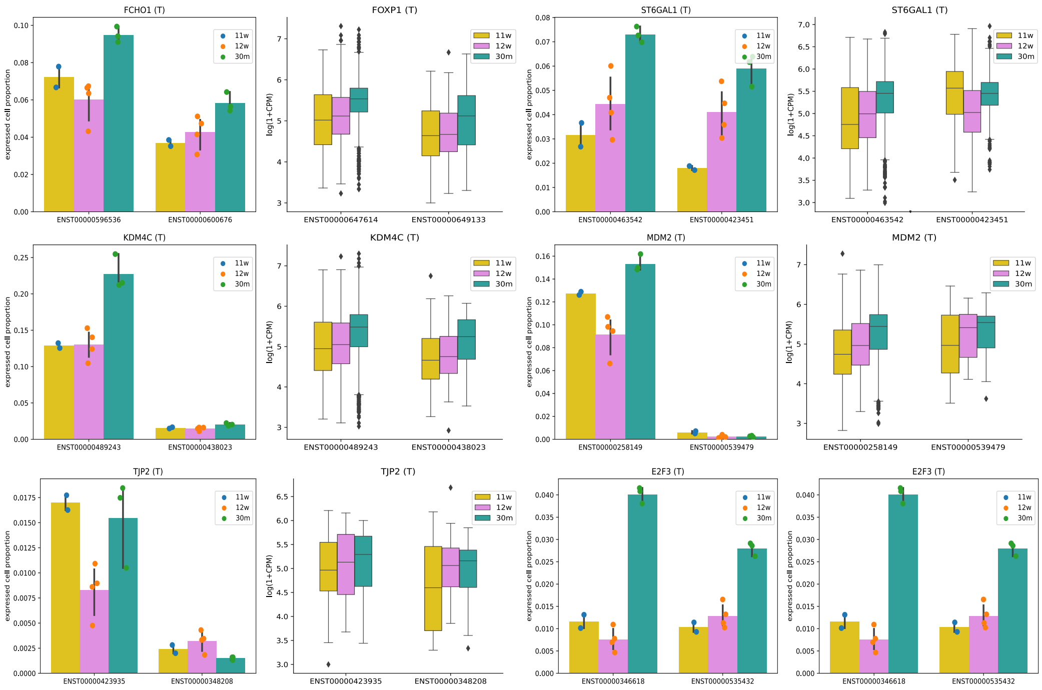

Figure S30: The expressed cell proportion and expression value of the example genes shown in Supp. Fig. S22.

A

window size	No. of CTSS	Percentage of annotated CTSS
15bp	2613	38.5%
30bp	2811	39.3%
60bp	2224	39.2%

B

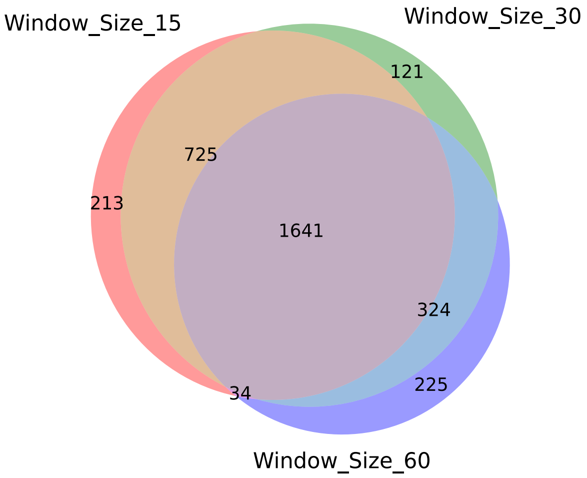

Figure S31: Evaluation for different window sizes including 15bp, 30bp and 60bp. (A). Statistic information for number of CTSS and percentage of annotated CTSS in different window sizes. (B). venn diagram showing the number of shared and unique CTSS among three window size groups.

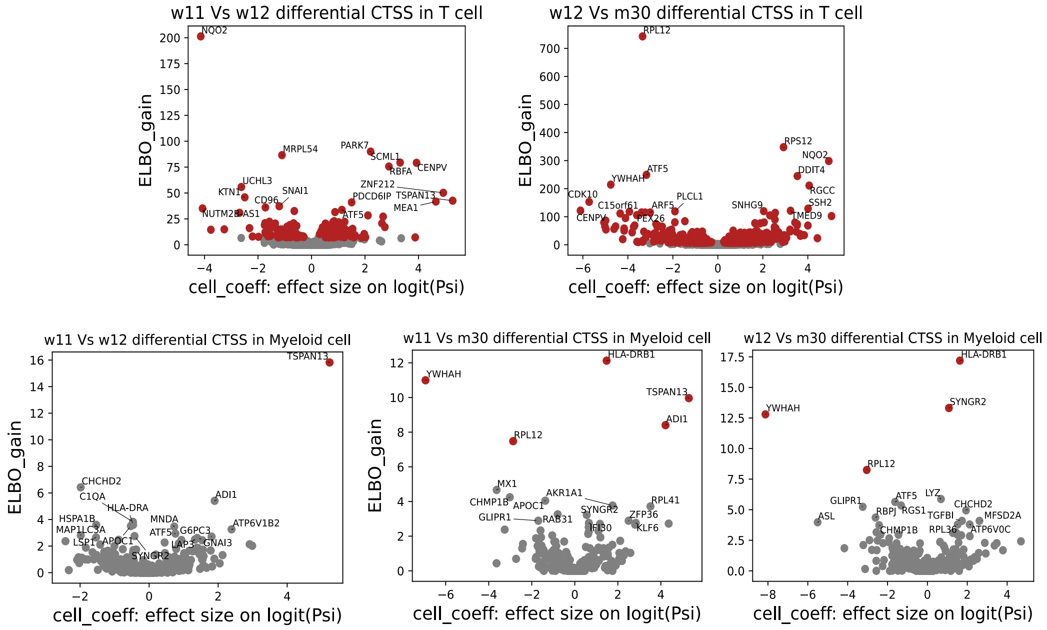

Figure S32: Volcano plots showing the relationship between ELBO_gain and effect size on logit(PSI) for differential CTSS between week11 and week12, week12 and month30 in T cells (Top) and in Myeloid cells (Bottom). The PSI value denotes the proportion of CTSS1 among the two major CTSSs. Source data are provided as a Source Data file.

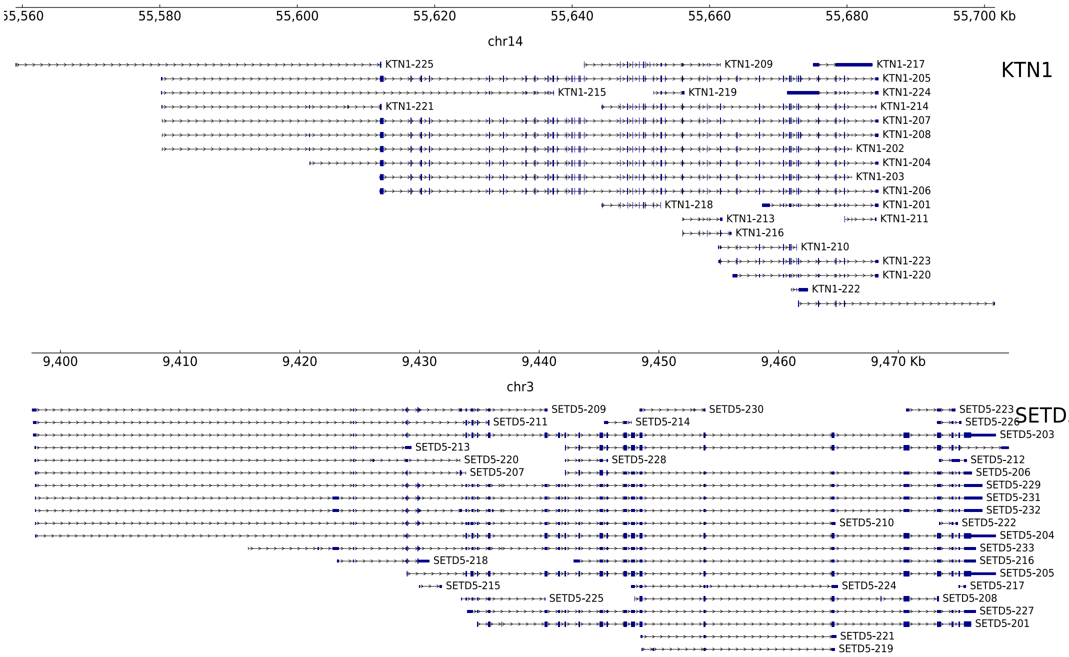

Figure S33: Track plots showing the structures and locations of TSS presented in the Fig. 5H (i.e. KTN1 and SETD5).

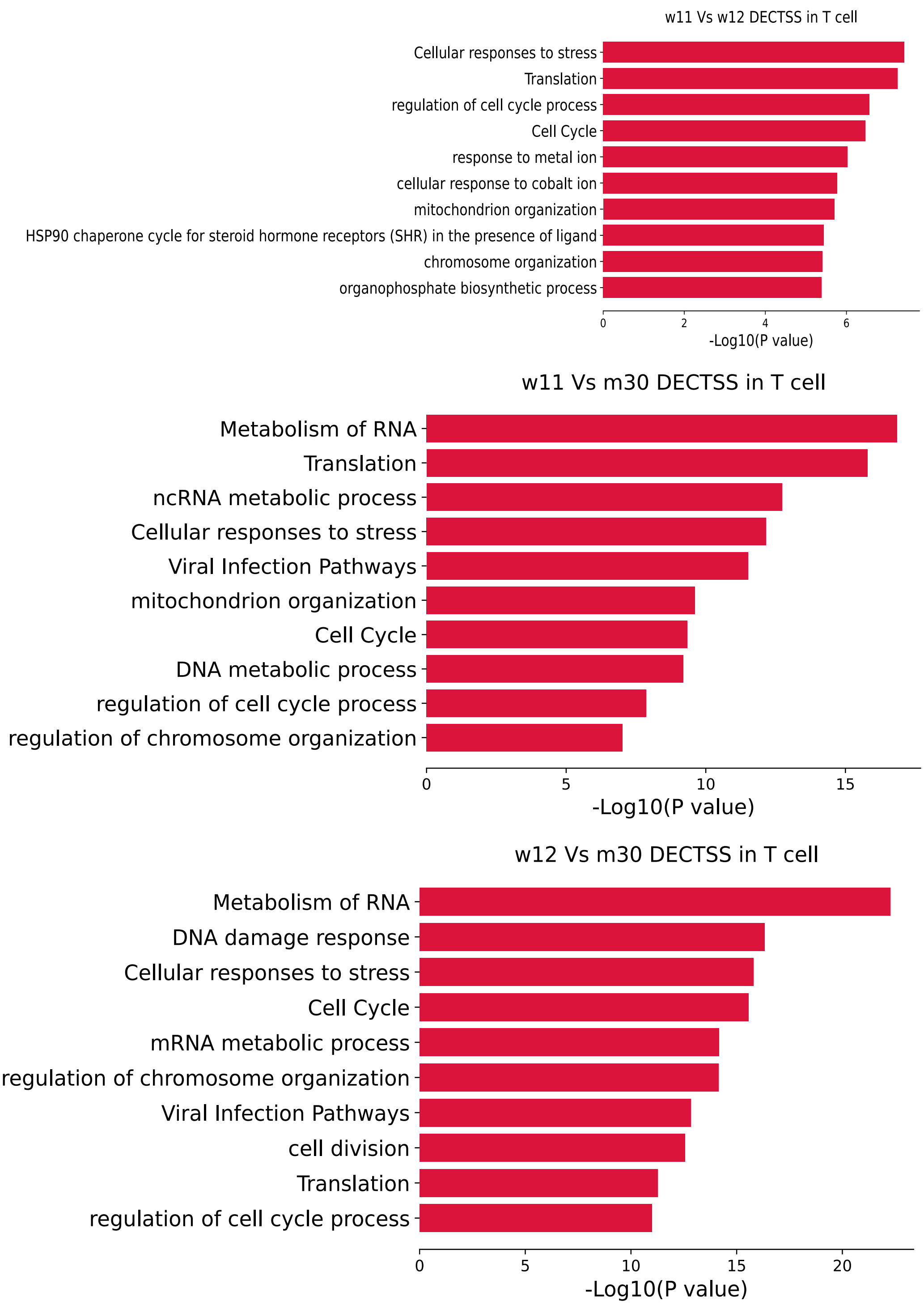

Figure S34: Bar graph of Gene Ontology enrichment analysis. Enrichment of gene ontology terms in gene list with a narrow shift within one TSS cluster respectively between 11-week and 12-week (top), 11-week and 30-month (middle), and 12-week and 30-month (bottom). Source data are provided as a Source Data file.
