# Supplementary material for: CamoTSS: analysis of alternative transcription start sites for cellular phenotypes and regulatory patterns from 5' scRNA-seq data
Source: Nat Commun. 2023 Nov 9;14:7240. doi: 10.1038/s41467-023-42636-1 (PMC10636040; doi:10.1038/s41467-023-42636-1)
Supplement: Supplementary file 4 — Description of Additional Supplementary Files [file 41467_2023_42636_MOESM4_ESM.pdf]

Description of Additional Supplementary Files:

Supplementary Dataset 1: Genes containing isoform markers masked at gene level in 15 organs

Supplementary Dataset 2: Genes with significant differential usage of alternative TSS between NPC and NLH in various cells.

Supplementary Dataset 3: Genes with significant differential usage of alternative TSS between normal and tumor epithelial cells in gastric cancer

Supplementary Dataset 4: The binding frequencies of human TF from JASPAR database with normal and tumor cells preferred TSS.

Supplementary Dataset 5: Genes with TSS shift across time points at the single cell level respectively between W11 vs W12, W11 vs M30 and W12 vs M30

Supplementary Dataset 6: Genes with TSS-shift in all three time points or just a certain development time point.

Supplementary Dataset 7: Genes of four modes including monotonic increasing and decreasing, TSS1 upregulation first and TSS1 downregulation first during human thymic development
